# Cross-species comparison of biological themes and underlying genes on a global gene expression scale in a mouse model of colorectal liver metastasis and in clinical specimens

**DOI:** 10.1186/1471-2164-9-448

**Published:** 2008-09-29

**Authors:** Obul Reddy Bandapalli, Christoph Kahlert, Victoria Hellstern, Luis Galindo, Peter Schirmacher, Jürgen Weitz, Karsten Brand

**Affiliations:** 1 Institute of Pathology, University of Heidelberg, Im Neuenheimer Feld 220/1, 69120 Heidelberg, Germany; 2 Department of Surgery, University of Heidelberg, Im Neuenheimer Feld 110, 69120 Heidelberg, Germany

## Abstract

**Background:**

Invasion-related genes over-expressed by tumor cells as well as by reacting host cells represent promising drug targets for anti-cancer therapy. Such candidate genes need to be validated in appropriate animal models.

**Results:**

This study examined the suitability of a murine model (CT26/Balb/C) of colorectal liver metastasis to represent clinical liver metastasis specimens using a global gene expression approach. Cross-species similarity was examined between pure liver, liver invasion, tumor invasion and pure tumor compartments through overlap of up-regulated genes and gene ontology (GO)-based biological themes on the level of single GO-terms and of condensed GO-term families. Three out of four GO-term families were conserved in a compartment-specific way between the species: secondary metabolism (liver), invasion (invasion front), and immune response (invasion front and liver). Among the individual GO-terms over-represented in the invasion compartments in both species were "extracellular matrix", "cell motility", "cell adhesion" and "antigen presentation" indicating that typical invasion related processes are operating in both species. This was reflected on the single gene level as well, as cross-species overlap of potential target genes over-expressed in the combined invasion front compartments reached up to 36.5%.

Generally, histopathology and gene expression correlated well as the highest single gene overlap was found to be 44% in syn-compartmental comparisons (liver versus liver) whereas cross-compartmental overlaps were much lower (e.g. liver versus tumor: 9.7%). However, single gene overlap was surprisingly high in some cross-compartmental comparisons (e.g. human liver invasion compartment and murine tumor invasion compartment: 9.0%) despite little histolopathologic similarity indicating that invasion relevant genes are not necessarily confined to histologically defined compartments.

**Conclusion:**

In summary, cross-species comparison on a global gene expression scale suggests the validity of an animal model representing the human situation. The actual yield of potential target genes depends on several variables including the animal model, choice of inclusion criteria, inherent species differences and histologic assessment.

## Background

Besides unrestricted proliferation and reduced apoptosis, unbalanced invasion is the third major prerequisite of malignant behaviour of the tumor cell. Invasion of tumor cells depends on a permissive host environment at the invasive site of the primary tumor as well as at the site of metastasis. The host participates in the induction, selection and expansion of neoplastic cells[1] to an extent that researchers are even raising the question of "who is invading whom?"[2]. Likewise, the tumor cells of the invasion front display features which differ from those in the inner parts of the tumor. We have recently reported on the host response of the liver tissue upon invasion by colorectal tumor cells as well as on the gene expression changes of invasive tumor cells in an immunodeficient murine xenograft model [3,4]. As part of our ongoing attempts to acquire cross-compartmental biological themes and to generalize findings obtained in distinct animal models with respect to the clinical situation, we now examined global gene expression in a syngenic immunocompetent mouse model and in a set of five clinical samples of colorectal liver metastases. We analyzed histology and global gene expression data from four compartments, namely liver, distant from the invasion front (L), liver adjacent to the invasion front (LI), tumor adjacent to the invasion front (TI) and tumor distant from the invasion front (T) and we particularly concentrated on the following three questions:

1. What is the degree of cross-species overlap on the single-gene level?

2. How similar are biological themes and single-gene expression data in a cross-species comparison and can relations between these parameters in addition to histological assessment be used to explain cross-species overlap?

3. Which biological themes and selected marker genes can be considered typical for the different compartments?

Our data indicate that cross-species overlap on the single-gene level depends strongly on the type of analysis but is generally sufficient to justify utility of the animal model. Analysis of gene expression based biological themes reveals that some findings on the single-cell level can be predicted by histopathology while others cannot. Thereby, ontologies provide a necessary biological bridge between standardized and routine methods of histopathologic assessment and single-gene expression analysis.

## Results

### 1. Intraspecies cross-compartmental correlation of histology and gene expression

Prior to cross-species comparisons, we wanted to examine within each species to what degree global gene expression changes correlate to the histological distinction of the four compartments: liver, liver invasion, tumor invasion and tumor. For this purpose, we compared the Affymetrix IDs (A+B chips covering the whole transcriptome) of each of the four compartments with each other on the gene expression scale separately for each species. By this procedure the number and percentage of differentially regulated IDs (increase calls plus decrease calls according to Affymetrix criteria) between each of the pairs of compartments was determined (Table 1). The comparison of tumor and tumor invasion compartments showed the lowest number of differentially regulated IDs, followed by the comparison of pure liver and liver invasion compartments (Table 1, upper panel). Comparison of compartments of different histological origin displayed higher values (Table 1, lower panel) than compartments of identical histologic origin. These data indicate that the fact of different histological origin (e.g. tumor versus liver) is reflected on the level of global gene expression and is not clouded by background noise in our model. On the other hand, the observation that there are differentially regulated IDs between L versus LI and T versus TI argues for a number of invasion specific processes in both species responsible for these differentially regulated IDs.

**Table 1:** Total number and percentage of Affymetrix IDs differentially regulated between any two compartments within one species (increase plus decrease calls)

	*Human*		*Murine*	
*Compartmental Comparisons*	*%*	*no*	*%*	*no*
				
*T/TI*	9.1 ± 0.1	1519 ± 9	22.5 ± 0.9	3452 ± 144
*L/LI*	23.8 ± 5.0	3785 ± 797	25.6 ± 3.5	3790 ± 519
				
*TI/LI*	28.8 ± 4.4	5310 ± 819	35.1 ± 4.2	5683 ± 675
*LI/T*	34.4 ± 4.8	5871 ± 80	28.6 ± 2.4	4327 ± 363
*L/TI*	37.8 ± 0.1	6817 ± 13	34.9 ± 0.9	5458 ± 138
*L/T*	40.8 ± 0.8	6555 ± 127	35.0 ± 0.9	5034 ± 124

100% is equivalent to all IDs present in at least one of the compartments to be compared.

L = liver, LI = liver invasion, TI = tumor invasion, T = tumor. Experiments in duplicates.

### 2. Cross-species overlap of compartment-specific up-regulated genes, GO-terms and histological similarity

We then wanted to determine the extent of cross-species overlap of up-regulated genes, which, in the invasion front, would represent potential target genes for tumor invasion. In addition, we wanted to evaluate whether these findings would be paralleled by the histopathology and gene expression based biological themes.

To determine single-gene overlaps, files of IDs were created, which included all genes present in one particular compartment within one species that display an "increase call" (up-regulation) as compared to each of the other three compartments of the same species (Atype-analysis). Subsequently, murine IDs were loaded into the NetAffyx program to obtain the list of corresponding genes. Then, using the "orthologues" function, murine genes were converted into their human counterparts. Overlaps of human genes from human tissue and converted genes from murine tissue were determined using a newly developed Excel Macro. Briefly, a colour code is assigned to each list of genes (murine converted into human and originally human), and genes are sorted alphabetically. The Macro will exclude any duplicate genes (several genes are represented by more than one ID) and count the number of unique genes in each group as well as the number of overlaps.

Biological themes (GO-terms) were determined using the Gene Ontology tree with its branches "biological process", molecular function" and "cellular component". Thereby, we defined those GO-terms which were "typical" for any of the 4 compartments. To obtain these compartment specific GO-terms, the following algorithm was applied:

1. Compartment specific up-regulated genes were selected as described above (test file).

2. A reference file was created including all genes which are present in at least one of the four compartments.

3. The distribution of the respective test files was compared to the distribution of the reference files in the GO-matrix (GOSSIP software).

To this extent, GO-term assignments (current versions available on ) as well as gene annotations (current version available on ) are loaded into the GOSSIP program to provide the matrix for comparison of ID-files. Then, a reference ID-file and a test ID-file are selected as described above and are loaded. The program will examine the distribution of IDs from test file and reference file among the GO-terms and assign a p-value to each GO-terms indicating the probability that a given GO-term is over-represented among the IDs of the test file respectively. In other words, the program examines how many IDs in the reference file and the test file belong to a given GO-term and compares the results from the test file and a reference file taking into account the total number of IDs in each file. Thus, a list of GO-terms is generated with high p-values indicating GO-terms typical (over-represented) for a given test file as compared to the reference file. To examine overlaps of GO-terms, pairs of GO-term-test files were compared using the Excel Macro as described above for comparison of single gene overlaps.

#### Syn-compartmental overlap (same compartments, different species, Table 2)

**Table 2:** A-E: Cross-species overlap of biological themes (GO-terms) and single genes expressed as absolute numbers (in brackets) or percentages of human genes or GO-terms overlapping with murine.

***A***	*Single Genes (A-Type Analysis)*			
***Murine***	*L*	*LI*	*TI*	*T*

***Human***				
	*% (487 ± 0)*	*% (356 ± 77.8)*	*% (642 ± 19.8)*	*% (378 ± 2.8)*
				
*L (491 ± 49.5)*	**27.9 **(137 ± *7*)	4.8 (23.5 ± *2.1*)	1.6 (8 ± 2.8)	1.2 (26 ± 1.4)
*LI (171 ± 1.0)*	9.9 (17 ± 4.2)	**3.5 **(6 ± *5.6*)	9.0 (15.5 ± 10.6)	4.7 (8 ± 2.8)
*TI(117 ± 9.9)*	2.6 (3 ± 1.4)	1.7 (2 ± *1.4*)	**13.2 **(15.5 ± 3.5)	8.2 (9.5 ± 0.7)
*T (202 ± 78.5)*	1 (2 ± 0)	2.8 (5 ± *0.7*)	6.7 (13.5 ± *6.3*)	**2.0 **(4 ± 2.8)

***B***	*Single Genes (B-Type Analysis)*			

***Murine***	*L*	*LI+TI*	*T*	

***Human***				
	*% (1649 ± 12)*	*% (3287 ± 216)*	*% (3089 ± 1220)*	
				
*L *(1228 ± 257)	**44 **(539 ± 50)	32 (394 ± 82)	9.3 (114 ± 42)	
*LI+TI (2412 ± 786)*	16.7 (403 ± 42)	**36.5**(880 ± 295)	26 (621 ± 194)	
*T *(2603 ± 555)	9.7 (252 ± 59)	40.7 (1060 ± 98)	34.6(902 ± 139)	

***C***	*Single Genes (C-Type Analysis)*			

***Murine***	*L*	*LI*	*TI*	*T*

***Human***				
	*% (7371 ± 490)*	*% (7909 ± 852)*	*% (8360 ± 238)*	*% (7216 ± 116)*
				
*L (491 ± 49.5)*	68.6(337 ± 36)	69.0 (339 ± 50)	59.7(293 ± 33)	51.5(253 ± 39)
*LI (171 ± 1.0)*	52.6(90 ± 53)	60.8 (104 ± 70)	60.8(104 ± 61)	55.5(95 ± 59)
*TI (117 ± 9.9)*	68.4(80 ± 7)	71.0 (83 ± 13)	79.5(93 ± 7)	71.0(83 ± 35)
*T (202 ± 78.5)*	37.1(75 ± 20)	42.0 (85 ± 21)	44.5(90 ± 36)	38.1(77 ± 29)

***D***	*Biological Themes (A-Type Analysis)*			

***Murine***	*L*	*LI*	*TI*	*T*

***Human***				
	*% (277 ± 2)*	*% (5 ± 4)*	*% (76 ± 26)*	*% (51 ± 1.4)*
				
*L (458 ± 42)*	**28.0 **(128 ± 7.8)	0.28 (1 ± 1.4)	2.0 (9 ± 6.4)	1.7 (8 ± 1.4)
*LI (133 ± 11)*	24.8 (33 ± 0)	**0 **(0 ± 0)	**16.5**(22 ± 1.4)	0.8 (1 ± 0)
*TI (46 ± 31)*	19.5 (9 ± 3.5)	0 (0 ± 0)	**10.9 **(5 ± 7)	10.9 (5 ± 1.4)
*T (11 ± 15)*	27.2 (3 ± 4)	0 (0 ± 0)	45.5 (5 ± 0)	**0 **(0 ± 0)

***E***	*Biological Themes (B-Type Analysis)*			

***Murine***	*L*	*LI+TI*	*T*	
***Human***				
		*% (315 ± 20.5)*		
				
*L*	nd		nd	
*LI+TI (97 ± 11.3)*		***53****(51.5 ± 5)*		
*T*	nd		nd	

The total number of up-regulated genes or over-represented GO-terms for each compartment is indicated in the first column (human) and the first line (murine) of each panel. Experiments in duplicates. Values of particular importance are in bold font (syn-compartmental comparisons) or underlined (cross-compartmental comparisons).

A-type analysis: Genes for interspecies comparison were selected according to the following criterion: Genes up-regulated in one particular compartment as compared to all other three compartments (truly compartment specific genes)

B-Type analysis: Criterion: Liver compartment: Up-regulated in liver as compared to tumor. Tumor compartment: Up-regulated in tumor as compared to liver. Liver invasion plus tumor invasion compartments: Combination of genes up-regulated in LI versus L and gene up-regulated in TI versus T.

C-type analysis: Criterion: Up-regulated in human compartments, present in murine compartments.

Cross-species syn-compartmental comparisons on the single gene level revealed that pure liver compartments showed the highest values among the species, namely 137 ± 7 overlapping single-genes (Table 2a, first column/first line). Tumor invasion showed the next highest values of 15.5 ± 11 genes (Table 2a, third column/third line). Liver invasion compartments (6 ± 5.6) and tumor compartments (4 ± 2.8) displayed very low overlap-values (Table 2a, second column/second line and fourth column/fourth line, respectively). Overlaps were similar if expressed as percentage of the number of up-regulated genes in human compartments (Table 2a).

Cross-species syn-compartmental comparisons on the level of GO-terms confirmed these data. Again, pure liver compartments showed the highest values among the species of 128 ± 8 overlapping GO-terms (Table 2d, fist column/first line). Tumor invasion showed the next highest values of 5 ± 7 (Table 2d, third column/third line). No GO-term overlaps were observed for liver invasion compartments and tumor compartments (Table 2d, second column/second line and fourth column/fourth line, respectively). Overlaps showed a similar tendency if expressed as percentage of the number of up-regulated genes in human compartments (Table 2d). These data indicate marked differences of cross-species similarity if compartments of the same histologic origin are compared according to the strict criteria of "up-regulation in one compartment as compared to all other compartments" (A-type analysis).

Since we reasoned that a number of liver-characteristic genes will not only be present in the pure liver compartments but in the liver invasion compartments as well, and that these genes will be consequently lost from further analysis by applying our strict criteria (A-type analysis), we relaxed criteria to obtain a true tissue specific comparison (B-type analysis): Liver specific genes were now defined as genes up-regulated in the liver compartment as compared to the tumor compartment only (not taking into account liver invasion or tumor invasion compartments). Cross species overlap between liver compartments was now found to be 44% on the single gene level (Table 2b, first column/first line). In a respective way, we reasoned that many tumor-characteristic genes will be excluded from further analysis because they will be present not only in the pure tumor compartment but in the tumor invasion compartment as well and therefore will not pass our strict criteria: Tumor specific genes were now defined as genes up-regulated in the tumor compartment as compared to the liver compartment only (not taking into account liver invasion or tumor invasion compartments). Applying this new criterion, cross species overlap between tumor compartments was now found to be 36.5% on the single gene level (Table 2b, third column/third line).

We examined yet another way of relaxing the cross species comparison: Overlap of exclusively up-regulated genes in one species with genes present in the other species was performed (C-type analysis). Cross-species overlap could be increased to more than two-thirds (68.6%, Table 2c, first column/first line) if exclusively up-regulated genes in human liver had only to fulfill the criteria of being "present" (but not necessarily "up-regulated") in the murine counterpart. Similarly, cross species overlap between tumor compartments approached 38.1% if exclusively up-regulated genes in human tumor were at least present (but not necessarily up-regulated) in the murine counterpart (Table 2c, fourth column/fourth line).

#### Cross-compartmental overlap (different compartments, different species, Table 2)

Cross compartmental overlap was generally lower than syn-compartmental overlap indicating that histopathologic similarity is paralleled by global gene expression. E.g. if overlap of tumor and liver genes was analysed, this resulted in low values if applying the strict criteria (A-type analysis) of 1.0% and 1.2% (Table 2a) and in still fairly low values of 9.7% or 9.3% if B-type analysis was performed (Table 2b).

As an important exception, cross-compartmental overlap was surprisingly high between human liver invasion and murine tumor invasion compartments on both, the single gene (15.5 ± 10.6) and the GO-term (22 ± 1.4) level (Table 2a and 2d, third column/second line), and this tendency was even more pronounced if values were expressed as percentages (Table 2a and 2d, second column/third line). This result indicates that genes involved in invasion may overlap between the species independent from histologically defined compartments. In order to further support this hypothesis, we examined the cross species overlap of genes characteristic for the invasion front as a whole by combining invasion front compartments (LI plus TI). To this extent, firstly, genes up-regulated in liver invasion compartments as compared to liver compartments and genes up-regulated in tumor invasion compartments as compared to tumor compartments were selected (B-type analysis). Then, these tumor invasion and liver invasion specific genes were combined to yield all invasion specific genes irrespective from liver or tumor origin. Subsequent analysis of cross-species overlap of these combined invasion front genes yielded a 36.5% overlap (Table 2b, second column, second line). The overlap on the level of biological themes even approached 53% (Table 2e, second column, second line).

Further relaxation of inclusion criteria by cross-compartmental comparisons of the "up-regulated versus present" = C-type analysis (Table 2c) yielded very high values indicating that compartmental specificity is lost by this relaxation of criteria.

Altogether, the data displayed in Table 2 indicate that for most compartments the majority of genes typical for one compartment will be found at least to be present in the respective compartment of the other species (Table 2c, C-type analysis). Still more than one third of genes characteristic for liver tissue, invasion front tissue or tumor tissue will be found to overlap with the respective compartments from the other species (Table 2b, B-type analysis). Less than one third of genes are found to overlap if truly compartment specific genes are compared (Table 2a, A-type analysis).

### 3. GO-terms and GO-term families

We next wanted to know which biological mechanisms were responsible for the observed cross-species similarities and gene expression overlaps. To this extent, the distribution of IDs among GO-terms was examined as described above. Biological themes as represented by GO-terms (biological processes, molecular functions and cellular compartments) fulfilling the following criteria were included into further analysis:

1. Number of underlying genes per GO-term >9

2. No terms of lower hierarchy clearly contributing to a significant higher node (e.g. exclusion of "collagen V" if "collagen" is significant)

3. Only one out of two or more alternate and significant terms (either "steroid binding" or "steroid metabolism")

4. No very general GO-terms of highest hierarchical levels (e.g. "membrane", "extracellular")

**Figure 1:**
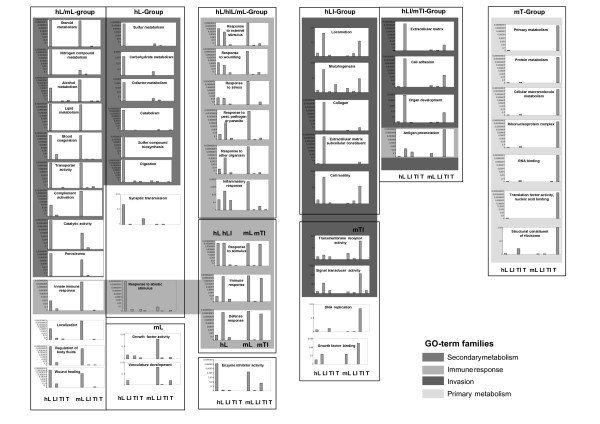
**Compartmental distribution of compartment specific over-represented GO-terms**. GO-terms were selected as described in the text. GO-term-diagrams are ordered in columns according to their compartmental distribution. The respective significant compartments are indicated in bold. Each diagram consists of 4 human compartments (to the left) and 4 murine compartments (to the right). Abbreviations: hL = human liver, hLI = human liver invasion, hTI = human tumor invasion, T = human tumor, suffix "m" indicates murine compartments respectively. Groups of GO-terms with identical compartmental distribution are framed. GO-term families are indicated by different grey scales (see insert). The GO-term "antigen presentation" belongs to the GO-term families "immune response" and "invasion".

Application of these criteria resulted in 53 "core" GO-terms (Figure 1). For each GO-term, a diagram was constructed based on the p-values for the four different compartments in both species (Figure 1). Such a diagram allows one to directly visualize in which compartment one particular GO-term is over-represented. Comparing the four bars to the left (human) with the ones to the right (murine) allows one to directly compare significant GO-terms between the two species. GO-term-diagrams displaying the same compartmental distributions were grouped and the statistically significant compartments were indicated (Figure 1, framed). In principle, the 8 examined compartments (2 species × 4 compartments) with significant p-values could be combined in 256 possible ways (all possible combinations of 2–8 compartments = binomial coefficient n over k = n!/(k!(n-k)!)). However, only 10 combinations were actually found, indicating a non at random, and probably biologically meaningful distribution of over-represented GO-terms among the different compartments. These 10 groups of GO-term diagrams with identical compartmental distribution were ordered into columns and were named according to the significant compartments (e.g. hL or hLI mTI, Figure 1, framed). Subsequently, GO-terms were grouped into GO-term families by biological reasoning. 44 out of 53 GO-terms were assigned to 4 larger GO-term families: Secondary metabolism, invasion, immune response and primary metabolism (Figure 1, different grey scales).

#### Secondary metabolism

The syn-compartmental cross-species evaluation revealed a huge number of GO-terms belonging to secondary metabolism among the liver compartments in both species (Figure 1, column 1, second darkest grey). In contrast, none of the other three compartments showed over-representation of any secondary metabolism term indicating that this GO-term family is liver specific. In addition, the human liver displayed some more metabolism terms that were not found to be statistically significant in the murine liver (Figure 1, column 1). Some additional terms, not belonging to the secondary metabolism family were found to be over-represented in murine and human livers separately or in combination.

#### Immune response

GO-terms positioned either upstream or downstream of the marker term "Immune response" were distributed along the liver, liver invasion and tumor invasion compartments in both species (Figure 1, second lightest grey). Only the tumor compartment did not show any significant "immune response" -related GO-terms. Different compartments seemed to be involved in innate and acquired immune response: The GO-terms "Innate immune response" (Figure 1) as well as "neutrophil activation" and "phagocytosis" (not shown) were over-represented in the liver compartments but not in any invasion front compartments. In contrast, the GO-terms "antigen presentation" (Figure 1), and "antigen processing" (not shown) typical for the acquired immune response were over-represented in the invasion front compartments in both species, but not in the liver compartments.

#### Invasion

GO-terms which have earlier been detected in a nude mouse model to be typical for invasion [3] were over-represented in both species in the invasion front compartments but not in any of the other compartments (Figure 1, darkest grey). Some terms such as "locomotion"/"cell motility" were over-represented in human liver invasion only, and others such as "signal transducer activity" were over-represented in murine tumor invasion only, while not reaching statistical significance in the other invasion compartments. Several GO-terms such as "extracellular matrix, "cell adhesion" and "differentiation"/"development" were over-represented in the human liver invasion compartment and in the murine tumor invasion compartment. This finding indicates that the invasion compartments, in addition to immune response GO-terms (see above), display typical invasion-related GO-terms. As expected from the previous findings, only the murine tumor compartment and the human liver compartment displayed over-represented invasion terms. This phenomenon may indicate a high degree of similarity of human and murine tumor invasion on the GO-term level, which, however, remains only true if the invasion front is examined as a whole.

#### Primary metabolism

In the murine but not in the human tumor compartment, a prominent "primary metabolism" cluster was found (Figure 1, lightest grey). This finding will be commented in the discussion section.

In summary, these data indicate that GO-terms can be condensed by applying an inclusion algorithm and by simple biological reasoning to reveal four GO-term families that include most GO-terms and that seem to represent expected biological functions, at least in liver and invasion front compartments.

### 4. Single-gene overlap within overlapping GO-terms

We finally wanted to examine, whether the observed degree of overlap on the single-gene level (Table 2b) would still hold true if the constitutive genes of actually overlapping GO-terms were compared between the species. In addition, compartment-specific up-regulation of overlapping genes was confirmed on the mRNA level by qPCR.

For the determination of single-gene overlaps within the "secondary metabolism" family we chose two main metabolism sub-terms that are particularly characteristic for liver function and at the same time comprise a high number of IDs: "lipid metabolism" and "nitric compound metabolism". As indicated in Table 3 (first panel), 16.1% of human "lipid metabolism" genes and 17.8% of human "nitrogen compound metabolism" genes were identical among the human and murine liver compartments.

**Table 3:** Cross-species overlap of single genes underlying specific GO-terms

	*Human*	*Murine*	*Overlap*	
	*No*.	*No*.	*No*.	*%*
***Secondary meatabolism***				
*Lipid metabolism*	59 ± 5.7	63.5 ± 2.1	9.5 ± 2.1	16.1
*Nitrogen compound metabolism*	53.5 ± 10.6	24 ± 1.4	9.5 ± 2.1	17.8
				
***Immune response***				
*Innate immune response*	10.5 ± 2.1	18 ± 0	5 ± 1.4	47.6
*Antigen presentation*	6 ± 0	12 ± 2.8	2 ± 0	33.3
				
***Invasion***				
*Cell Adhesion*	23 ± 4.2	40.5 ± 3.5	2 ± 1.4	8.4
*Extracellular Matrix*	20 ± 4.2	21.5 ± 2.1	3 ± 1.4	15

Upper three GO-terms from both liver compartments, lower three GO-terms from human liver invasion and murine tumor invasion. Overlaps as total number and in percent of human genes. Experiments in duplicates.

Compartment-specific up-regulation was verified on the arbitrarily selected *apolipoprotein F *gene from the "lipid metabolism" GO-term. The gene showed the highest levels of mRNA in the liver compartment in both species (Table 4). Gene activity decreased with increasing distance from the liver compartment in both species.

**Table 4:** Gene expression of selected genes typical for specific compartments

*Gene Name*	*GO-term*		*L*	*LI*	*TI*	*T*
*Apolipoprotein F*	*Lipid metabolism*	*Human*	**100%**	41.75%	17.4%	16.1%
		*Murine*	**100%**	26.5%	22%	7.9%
						
*Thrombospondin-2*	*Cell adhesion*	*Human*	21.6%	**100%**	22.7%	8.2%
		*Murine*	4%	7.2%	**100%**	18.1%
						
*Procollagen V-alpha 2*	*Extracellular matrix*	*Human*	12%	**100%**	73.5%	28.7%
		*Murine*	4%	20.6%	**100%**	14%

RNA-levels in percent of gene expression of the compartment with highest RNA levels (= 100%). Experiments in triplicates.

For the determination of single-gene overlaps within the "immune response" family we chose the two sole immune response terms: "innate immune response" and "antigen presentation" present in the hL/mL group and the hLI/mTI group respectively (Figure 1). As shown in Table 3 (second panel), 47.6% of human "innate immune response" genes were identical among the human and murine liver compartments and 33.3% of human "antigen presentation" genes were identical among the human liver invasion compartment and murine tumor invasion compartment.

For the determination of single-gene overlaps within the "invasion" family we chose arbitrarily among the terms that we have earlier found to be characteristic for invasion in another mouse model: "cell adhesion" and "extracellular matrix". As shown in Table 3 (third panel), 8.4% of human "cell adhesion" genes and 15% of human "extracellular matrix" genes were identical among the human liver invasion compartment and the murine tumor invasion compartments.

Compartment-specific up-regulation was verified on arbitrarily selected genes from the "cell adhesion" and the "extracellular matrix" GO-terms. Both genes, *thrombospondin-2 *and *procollagen V-alpha 2 *showed the highest level of mRNA in the human liver invasion compartment and the murine tumor invasion compartment respectively (Table 4).

We did not determine single-gene overlap of any "primary metabolism" GO-terms because the GO-terms did not reach statistical significance in any of the other compartments.

Altogether, these data indicate that single genes underlying overlapping GO-terms display cross-species identity to a varying degree. In addition, up-regulation on microarrays was validated by independent qPCR on arbitrarily selected genes.

## Discussion

Murine models represent a necessary tool in cancer research. However, there is always uncertainty about the extent to which findings in the animal can be related to the human situation. In this study we examined whether global gene expression profiling in addition to standard histopathologic examination can assist in judging on the suitability of a murine model of colorectal liver metastases for the detection of invasion front target genes.

As a result from our study, it appears that the utilized animal model (CT26/Balb/C) represents the clinical situation to an extent that will allow successful mining of target genes. Among the potential target genes in the invasion front are *thrombospondin-2 *and *procollagen V-alpha 2 *that were confirmed with semi-quantitative real time PCR. Among gene ontology derived biological processes and molecular functions typical for invasion front were "extracellular matrix", "cell motility", "cell adhesion" and "antigen presentation". The same or similar GO-terms or underlying genes were found to be overrepresented in invasion front compartments in a nude mouse xenograft model of colorectal liver metastases as previously reported by us [3] as well as in other studies on clinical colorectal specimen [5,6] and specimen from other tumor entities [7,8] indicating that the invasion front compartment indeed constitutes a biologically defined compartment.

However, a number of variables seem to influence the extent of cross-species overlap and the yield of potential target genes which requires further discussion:

### 1. Selection criteria

The number of potential target genes strongly depends on the criteria that are used for data acquisition: If only genes are compared which are specifically up-regulated in one particular compartment as compared to all other three compartments (A-type analysis) only 21.5 (5.6 (LI/LI) + 15.5 (TI/TI), Table 2a) overlapping potential target genes (out of more than 45000 IDs on each chip) are obtained. However, if we include cross-compartmental overlaps (15.5 (LI/TI) + 2 (TI/LI) = 17.5) we obtain altogether twice as many genes (21.5 syn-compartmental + 17.5 cross-compartmental = 39). It should be mentioned that the prominent cross compartmental overlaps become only evident by the analysis of biological themes (Table 2d and 2e) which consequently prompted us to combine histologically distinct compartments. Despite different tissue of origin, typical invasion front GO-terms such as "cell adhesion", "extracellular matrix", "organ development" and "antigen presentation" were present in human liver invasion but in murine tumor invasion, which indicates that these compartments may be functionally similar. Most of these genes are probably excellent candidate target genes and some of them were validated by qPCR (Table 4).

However, we still felt that many potential target genes may be lost by inappropriate strictness of criteria. Consequently, we alleviated criteria further by defining invasion front specific genes as those genes up-regulated in invasion front compartments as compared to their normal counterparts only (e.g. liver invasion as compared to pure liver, B-type analysis) instead of requiring up-regulation as compared to all other compartments (A-type analysis). This modification of inclusion criteria led to a huge increase of up-regulated genes. If subsequently, genes up-regulated in both invasion front compartments (TI+LI) were combined this resulted in an impressive overlap of 880 potential target genes. From these data we conclude that modifications of inclusion criteria may result in very different yields of potential target genes (47 versus 880 genes). Importantly, examination of overlapping genes and biological themes (GO-terms) resulted in similar GO-terms families in B-type analysis followed by combination of TI and LI compartments (data not shown) as obtained for A-type analysis (Figure 1). This indicates that invasion front specificity is retained and higher overlapping values are not the result of an increase of unspecific background. Although, we have no definite explanation for these differences in yield, we assume that many genes typical for the invasion front as a whole will be excluded from further analysis by applying Atype analysis: In particular, those genes which are of similar intensity in the TI and the LI compartments within each species will be lost upon A-type analysis which requires up-regulation in any compartment as compared to all other three compartment. In contrast, these genes will be included in B-type analysis because they will be up-regulated either in LI versus L or in TI versus T.

On the other hand, alleviation of criteria has to be performed with caution. If we only require that specifically up-regulated genes in one particular compartment have to be present (but not necessarily up-regulated, C-type analysis) in the respective compartment of the other species, again a huge increase of target genes results (104 (LI/LI) + 104 (LI/TI) + 83 (TI/LI) + 93 (TI/TI) = 384, Table 2c). However, equally high values are obtained if tissues of different origin like liver and tumor are compared (253 (L/T) + 75 (T/L) = 328, Table 2c) indicating a low level of specificity and accordingly many false positive genes that are actually not suitable as potential target genes.

### 2. Inherent species differences

Differences of liver tissue compartments far from the invasion front may represent the degree of dissimilarity between the species of only marginally affected organs. Our data indicate that the biology of human livers as compared to the livers of Balb/C mice is not exactly the same as only 27.9% of up-regulated genes were found to be overlapping in A-type analysis and still only 44% of genes overlap resulting from B-type analysis. It is likely that these inherent dissimilarities of the host tissue will have a severe impact on the mechanisms in the invasion front.

Although this present approach is to our knowledge the first whole genome cross-species approach for invasion, several other cross-species comparisons on a more or less genome- wide scale have been performed. It is however difficult to compare data due to differences in methods and models. Sometimes, an encouraging similarity between the species is reported such as in a microarray meta-analysis of the complex biological phenotype of aging. Here, the authors identified an expression signature common to the aging transcriptomes of mouse, man and rat at least within some organs [9]. A nice correlation of gene expression data from a rat liver cancer model with clinical cytogenetic aberration profiles resulted in the identification of several pathways involved in human liver cancer [10]. A general similarity of biological themes as well as on region specific genes has been observed upon the comparison of human and murine healthy brains ([11] and references herein). Similar gene expression profiles were also found in a comparison of baseline and selenium treated rat and human prostate tissues [12]. Results from a multi-species gene expression profiling approach (dogs, rats, mice, human cells) on the response on ventilator-associated lung injury suggested the feasibility of such an approach for the evaluation of biological processes of interest and selection of process-related candidate genes [13]. In contrast, even on the level of one single-cell type in vitro, marked cross-species differences have been observed in a massive parallel signature-sequencing approach, which identified only a small (core) set of conserved genes between human and murine embryonic stem cells [14]. These data indicate that, depending on the biological context, the animal model can be closer or more deviant from the clinical situation.

### 3. Tumor model

Only 2% of exclusively up-regulated genes were overlapping among the tumor compartments in A-type analysis (Table 2a), and no overlapping GO-terms between the tumor compartments were detected (Table 2d), which obviously has a severe impact on the mechanisms in the invasion front. The percentage of overlapping genes was dramatically increased to 34.6% if the criteria of B-type analysis were applied (Table 2b) but is still far from 100%. Interestingly, the murine tumor displayed more features of uniqueness than its human counterpart (51 vs. 11 GO-terms, data not shown). Most of these terms were associated with primary metabolism. In contrast to secondary metabolism (characteristic for liver) that produces and breaks down compounds that are essential for the whole organism, primary metabolism contains all pathways necessary to keep the cell alive. While the liver is known to be the classical site of secondary metabolism (see above), it is more difficult to explain why only the murine tumor compartment displays such a huge number of primary metabolism terms. Primary metabolism is also defined as normal anabolic and catabolic processes which result in assimilation, respiration, transport, and differentiation and which directly function in the processes of growth and development. A closer look at the sub-terms reveals that several belong to protein biosynthesis so that we have concluded that the tumor compartment in the murine model is mainly occupied with maintaining its primary functions represented e.g. protein biosynthesis. Histologically, both examined cancer types were adenocarcinomas of colorectal origin. However, the murine tumor is a lowly differentiated to undifferentiated carcinoma whereas the human specimens manifest moderate to low differentiation. These histological differences may partly explain low overlap of tumor compartments.

### 4. Misleading histology

Another reason why we observed limited single-gene overlaps in the invasion front compartments on A-type analysis may be due to the fact that mechanisms involved in invasion do not appear to respect boundaries as estimated by standard histopathologic observation. The most striking observation was the relatively high degree of overlap between human liver invasion and murine tumor invasion which was 9% (versus 3.5% with murine liver invasion) on the single-gene level and 16.5% (vs. 0% for murine liver invasion) on the GO-term level. As a second example, liver compartments displayed strong "immune response" GO-terms that may be a part of a host counterattack against the invading tumor (Figure 1). Liver immune response consisted mainly of innate immune response whereas invasion-related immune response was characterized by acquired immune response.

From these data we conclude that when one is mining for invasion target genes, one should consider the invasion front as a whole (as discussed in the first paragraph) and even take tissue further away from the invasion front like the liver compartments into consideration.

### 5. Functional redundancy

A fifth reason for the limited overlap of target genes is due to the redundancy of gene regulation within biological processes. Neoplastic processes that are targeted by drugs include proliferation, apoptosis, invasion and several more. These processes are on the descriptive level of biological themes. Many animal models for target gene determination or validation are chosen according to whether they exhibit these biological theme-like functions. As our data show, the degree of similarity on the level of general biological themes (GO-term families) between clinical samples and the animal model can be fairly high, but it decreases dramatically with an increase of specificity of the underlying processes as seen by the lower overlap of single GO- terms and single genes.

The liver compartments in our model, for example, displayed a high degree of cross-species, syn-compartmental similarity on the level of GO-term families. E.g. from the 15 core GO-terms of the "secondary metabolism" family, 9 GO-terms were over-represented in the liver compartments in both species whereas only 6 core GO-terms were species-specifically over-represented in human liver only (Figure 1, columns 1 and 2). In contrast, if GO-terms closer to the small branches of the GO-tree, usually covering less than 10 genes, the syn-compartmental, cross-species overlap was only 28% (Table 2d). Finally, overlap on the single-gene level (all genes) was only 27.9% as well, and the degree of overlap of the genes underlying overlapping GO-terms was even lower (16.1% for the "lipid metabolism" term, Table 3). In other words, although the liver compartments apparently carry out typical liver functions related to secondary metabolism in both species and even sub-terms like "lipid metabolism" are significantly over-represented in the liver of both species, only a minor portion of the actual underlying genes are indeed identical. Due to reasons described above, the situation is even more complicated for invasion front and tumor compartments. That ultimately means that targeted biological processes are by nature represented by a variety of perhaps similar, perhaps alternative or even redundant genes.

The problem of functional redundancy can probably be circumvented if the animal model is chosen *a priori *to have the same molecular defect as the human counterpart tumor. A recent publication argues in favour of this approach. A good correlation of gene expression profiles for a mouse model of KRAS2-induced lung cancer and KRAS2-mutated human lung carcinoma was reported [15].

It is notable that in that same study [15], the gene expression signature of KRAS2 activation was not identifiable by analysis of human tumors alone, but only by integration of mouse and human data. This integration indicates that a murine model, in addition to displaying molecular similarity, could uncover biological themes or pathways relevant to human cancer that are obscured in the human data. Similarly in our model, only through cross-species gene expression profiling, we uncovered the high degree of unspecific immune response in the liver away from the invasion front, which would probably not have gained attention if only one species would have been used.

## Conclusion

In summary, histology and gene expression based analysis of biological themes are valuable tools to understand cancer- relevant processes and to judge on the suitability of animal models. However, due to inherent species differences and functional redundancy, the number of actual target genes that are similarly regulated in the clinical situation and the animal model has to be determined individually in standard grafted models and is usually far below 100%. The outcome of interspecies comparisons on a global gene expression scale is further dependent of a considerate use of a selection criteria and histologic assessment.

## Methods

### Cell lines and animals

Liver metastases in Balb/C mice (n = 5, Möllegard und Bomholdgard Laboratories, Ry, Denmark) were induced by intrasplenic injections of CT-26 murine colon adenocarcinoma cells [16] as previously described [3]. The model produces extensive liver colonization (more than 20 deposits per animal liver) after a period of 2 to 4 weeks. At that time, the animals were euthanized, livers from 5 animals were removed and the material was processed for microdissection as previously described [3,4].

### Clinical specimen

Tissue was collected with informed consent of all patients (n = 5). Tissue collection was approved by the University of Heidelberg ethics committee.

### Tissue preparation and laser microdissection (LMD)

**Figure 2:**
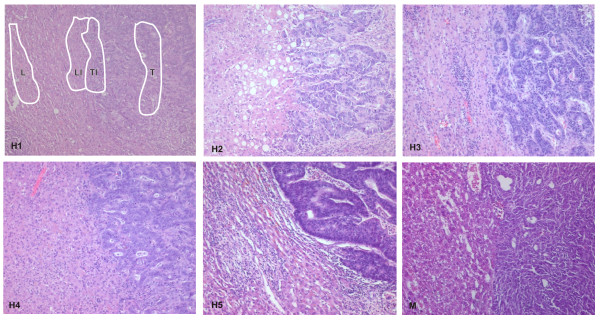
**Histology of invasion fronts of liver metastases from the clinical specimen and from the murine model**. HE staining of the invasion front of five clinical specimens (H1–H5) and murine tumors (M, CT26) growing in the livers of Balb/C mice. The human tumors were moderately to lowly differentiated adenocarcinomas with moderate stroma production and stroma reaction by a mild lymphocytic infiltrate. The tumor part of the invasion front did not differ strikingly from the inner parts. The liver displays an orderly structure with some fibrosis and a mild to moderate lymphocytic infiltrate in the portal tracts, no major pigments and mild to moderate fatty degeneration. The liver part of the invasion front contains mainly hepatocytes, but, in addition, an increased number of inflammatory and fibroblast like cells as well as sporadically ECM-like deposits. The murine tumor is a lowly differentiated to undifferentiated adenocarcinoma with little stroma production and little stromal reaction. As in the clinical samples the tumor does not significantly differ from the tumor part of the invasion front. The livers are of orderly structure with no apparent abnormalities. The liver part of the invasion front does not display gross differences to the liver except that the hepatocytes appear to be slightly flattened. Areas of microdissection are displayed exemplarily (H1).

Frozen tissue blocks were cut into 15 μm sections using a cryostat (Leica, Wetzlar, Germany) and stained using cresyl violet according to the Ambion (Austin, TX, USA) LCM staining kit protocol. Four distinct cell populations were separately microdissected with LCM equipment (Molecular Machines & Industries, Eching, Germany or PALM, Bernried, Germany): a) pure liver tissue at least 10 rows away from the invasion front, b) liver invasion front tissue extending 5 cell rows into the liver, c) tumor invasion front tissue extending 5 cell rows into the tumor and d) pure tumor tissue at least 10 rows away from the invasion front (Figure 2, H1). These compartments were arbitrarily selected to due prior experience [4] and results from immunostaining of up-regulated genes (unpublished data). Microdissection was performed separately by two different scientists yielding material for human and mouse experiments.

### RNA amplification

Total RNA from microdissected samples was extracted (RNeasy Mini Kit, Qiagen, Hilden, Germany) and quality was evaluated by using an Agilent (Waldbronn, Germany) 2100 Bioanalyzer. For murine metastases, 30 ng of RNA corresponding to 2500–3500 cells from each microdissected group were amplified (RiboAmp HS RNA Amplification Kit, Arcturus, Sunnyvale, CA, USA), labelled and the resulting biotinylated cRNA targets were used to probe the GeneChip Mouse Expression Set 430 (A+B) (Affymetrix, Santa Clara, CA) as previously described [3,4]. For human metastases, 250 ng RNA corresponding to 20000–30000 cells from five clinical specimens were pooled, amplified (Message Amp Biotion Kit, Ambion) and probed on the Human Genome U133 set (A+B). Hybridisation was performed in duplicates. Altogether, 32 chips were hybridized (4 compartments × 2 sub-chips (A+B) × 2 species × 2 (duplicates) = 32).

### Data analysis

The scanned images from the chips were processed using Affymetrix GCOS and Excel (Microsoft, Seattle, USA) software. Statistical significance analysis of compartment-specific over-representation of GO-terms was performed with the GOSSIP program (Microdiscovery, Berlin, Germany [17]). For the determination of single-gene and GO-term overlaps, Netaffyx tools from the Affymetrix website ("orthologues" function, ) and a newly developed Excel macro were applied.

### Relative quantitative real time PCR

Microdissection and RNA isolation for relative qPCR were essentially performed as for hybridization experiments, however independent samples were used. 3 ng of total RNA, corresponding to 2500–3500 cells were used for quantification. Reverse transcription, qPCR, normalization (on 18S RNA) and efficiency correction (on 18S RNA) were performed essentially as described in [3]. Oligonucleotides for qPCR were designed using the Primer3 software (Whitehead Institute, Cambridge, MA, USA). The sequences for 18s RNA were: forward primer: 5'- AAA CGG CTA CCA CAT CCA AG -3', reverse primer: 5'- CCT CCA ATG GAT CCT CGT TA -3', for human *apoplipoprotein F *(gi:50659075) were: forward primer: 5'- TTC TGC ACC CAA AGT CAC TG -3', reverse primer: 5'- ATC AGC CTG ACA ACC AGC TT -3', for murine *apoplipoprotein F *(gi:19527215) forward primer: 5'- ATA CAG CCC AGC CGT CTA AA -3', reverse primer: 5'- CCA GGG ACA GAA AGG TTC AA -3', for human *thrombospondin-2 *(gi:40317627): forward primer: 5'- TAT TCC CGA GAC CAA CGA AG-3', reverse primer: 5'- ACA TCA TCG TCA CTC CCA CA-3', for murine *thrombospondin-2 *(gi:6755778): forward primer: 5'- GGG ACC ACA CAA ATT GAT CC -3', reverse primer: 5'-CCC AAA CTC GTC GAA ACC TA -3', for human *procollagen, type V, alpha 2 *(gi:86613789): forward primer: 5'- ACACACGTGCCCAGTAATGA-3', reverse primer: 5'-GGAAATCTATCCCAGCTTGC-3', and murine *procollagen*, *type V*, *alpha 2 *(gi|:89363016): forward primer: 5'- TGGAGAAGGTGGAAAACCAG -3', reverse primer:, 5'- TCTCCTCTTTCCCCAGGATT -3'.

### Statistics

The statistical significance of differential representation of GO-terms between test group and reference group was analyzed by the GOSSIP software [17], which uses Fisher's exact test corrected for multiple testing effects. Single-test p-values are included for completeness whenever the term was significant in at least one experiment after multiple testing corrections (FDR, false discovery rate).

## Authors' contributions

ORB performed microarray hybridizations, isolated RNA, performed qPCR and microarray data analysis. CK performed microarray hybridizations & RNA isolation. KB supervised the experiments and performed microarray data analysis. VH performed microarray data analysis. KB and ORB wrote the manuscript. LG, PS, and JW participated in the design of the experiments and provided clinical specimen.
